# Sexual selection constrains the body mass of male but not female mice

**DOI:** 10.1002/ece3.2753

**Published:** 2017-01-27

**Authors:** James S. Ruff, Douglas H. Cornwall, Linda C. Morrison, Joseph W. Cauceglia, Adam C. Nelson, Shannon M. Gaukler, Shawn Meagher, Lara S. Carroll, Wayne K. Potts

**Affiliations:** ^1^ Department of BiologyUniversity of UtahSalt Lake CityUTUSA; ^2^ Department of Molecular and Cellular BiologyHarvard UniversityCambridgeMAUSA; ^3^ Environmental Stewardship GroupLos Alamos National LaboratoryLos AlamosNMUSA; ^4^ Department of Biological SciencesWestern Illinois UniversityMacombILUSA; ^5^ Department of Ophthalmology and Visual SciencesUniversity of UtahSalt Lake CityUTUSA

**Keywords:** fecundity, intrasexual selection, mammals, sexual selection, sexual size dimorphism, stabilizing selection

## Abstract

Sexual size dimorphism results when female and male body size is influenced differently by natural and sexual selection. Typically, in polygynous species larger male body size is thought to be favored in competition for mates and constraints on maximal body size are due to countervailing natural selection on either sex; however, it has been postulated that sexual selection itself may result in stabilizing selection at an optimal mass. Here we test this hypothesis by retrospectively assessing the influence of body mass, one metric of body size, on the fitness of 113 wild‐derived house mice *(Mus musculus*) residing within ten replicate semi‐natural enclosures from previous studies conducted by our laboratory. Enclosures possess similar levels of sexual selection, but relaxed natural selection, relative to natural systems. Heavier females produced more offspring, while males of intermediate mass had the highest fitness. Female results suggest that some aspect of natural selection, absent from enclosures, acts to decrease their body mass, while the upper and lower boundaries of male mass are constrained by sexual selection.

## 1 Introduction

Body size is influenced by natural and sexual selection with both female‐ and male‐biased sexual size dimorphism (SSD), as well as monomorphism, common across vertebrates (Andersson, 1994). Selective forces for increased female size include a positive relationship with fecundity, enhanced resources for parental care, and dominance over resources, while those for decreased female size include increased maturation rate and decreased energy demands; conversely, male‐biased SSD is primarily driven by physical competition for mates with the largest individuals having the highest fitness (Andersson, 1994; Clutton‐Brock, 2009; Cluttonbrock & Parker, 1992; Schulte‐Hostedde, 2007). Taken together, fecundity selection in females and sexual selection in males are largely thought to be the primary selective forces driving larger body size across organisms; however, it has proven more difficult to understand the counteracting selection which constrains body size.

Blanckenhorn (2000) suggested four costs due to larger body size: (1) viability costs in juveniles due to longer development (or faster growth); (2) viability costs in adults due to predation, parasitism, or starvation; (3) decreased mating success of large males due to lack of agility or high energy costs; and (4) decreased fitness in both sexes due to late reproduction associated with longer development. These four hypotheses include pressures due to both natural (1 and 2) and sexual (3 and 4) selection; however, supporting evidence in vertebrates has been difficult to obtain for the two sexual selection hypotheses. Specifically, within vertebrates, costs associated with relatively large body size, in the context of male sexual selection, have only been demonstrated in the pied flycatcher (*Ficedula hypoleuca*) and serrate‐legged small treefrogs (*Philautus odontarsus*) (Alato & Lundberg, 1986; Zhu et al., 2016).

House mouse (*Mus musculus*) populations inhabiting semi‐natural enclosures are well suited for quantifying selective forces operating on a variety of phenotypes and provide a unique opportunity to assess the natural and sexual selective forces that constrain body size (Carroll & Potts, 2007). Within these enclosures some, but not all, pressures of natural selection (e.g., predation) are absent, and most sexual selection pressures are present (including male–male competition and female choice ((Meagher, Penn, & Potts, 2000; Nelson, Colson, Harmon, & Potts, 2013))). Therefore, by assessing the reproductive success of mice in semi‐natural enclosures, one can evaluate a trait's influence on fitness in the context of moderate levels of natural selection and high levels of sexual selection.

Here we assess the relationship between body mass (a measure of body size) and fitness in both sexes of house mice. Due to the nature of our study we control for three of the four hypothesized selective pressures on body size (1, 2, and 4 above) allowing us to assess whether male sexual section might act to constrain body size with counteracting pressures on males that are too small as well as those who are too large. We do this by retrospectively analyzing parentage and body mass data from three previous studies using our mouse semi‐natural enclosure system. Each of these studies directly tested outbred control mice in direct competition with experimentally manipulated mice; only control mice are analyzed here. The first study (S1) assessed the fitness consequences of inbreeding; parentage was conducted subsequently to evaluate the deleterious nature of the *t*‐complex (Carroll, Meagher, Morrison, Penn, & Potts, 2004; Meagher et al., 2000). The second (S2) and third (S3) studies assessed fitness consequences of pharmaceuticals (Gaukler et al., 2015, 2016). Collectively, the fitness and body mass data from these studies provide a unique opportunity to test the selective pressures that may constrain body mass in vertebrates.

## 2 Materials and methods

### 2.1 Animals

From 55 litters, 113 (75 female and 38 male) outbred wild‐derived mice were assessed. Mice from S1 (*n* = 77) were from the second generation of a colony initially described by Meagher et al. (2000), while those in S2 (*n* = 24) and S3 (*n* = 12) were from the twelfth. Mice entered enclosures as sexually mature adults (S1: 23.0 ± 9.5 weeks old, S2: 26.2 ± 7.1, S3: 27.1 ± 2.3, mean ± SD) and were weighed prior to release. Ten populations (S1: *n* = 7, S2: *n* = 2, S3: *n* = 1) were established with 16 females and eight males, half of whom were controls, and seven mice were not weighed. Collectively, these populations represent all published accounts from our laboratory with complete body mass and parentage data. The assessed studies were approved by the Institutional Care and Use Committee at the University of Utah (protocol #s 97‐11011, 07‐8002, and 10‐08002).

### 2.2 Semi‐natural enclosures

Indoor enclosures are 30–50 m^2^ and are subdivided to promote territory formation. Subsections have food and water provided ad libitum associated with nest‐boxes in either “optimal” territories (with enclosed nest‐boxes) or “suboptimal” territories (with exposed boxes). Photographs and diagrams of enclosures may be found in the initial studies (Gaukler et al., 2015, 2016; Meagher et al., 2000). Offspring born within S1 populations were removed at ~6.4 weeks of age, while in S2 and S3 all offspring were collected at eight weeks into the study and then again at five‐week intervals; after removal, offspring were euthanized and tissues were harvested. Populations were maintained for 30.0 ± 4.3 weeks.

### 2.3 Parentage

Four–17 autosomal microsatellite loci were amplified per offspring. Primers were tagged with CY‐5 or CY‐3 fluorescent dye. DNA samples were PCR‐amplified and run on 6.25% denaturing acrylamide gel at 40 W for 3–7 hours. Gels were imaged on a FluorImager. Additional details on parentage analysis, including loci used, can be found in original reports (Carroll et al., 2004; Gaukler et al., 2015, 2016).

### 2.4 Data analysis

For an initial approach, offspring counts of both sexes were first modeled together using a generalized linear mixed model (GLMM) with a Poisson distribution and logarithmic link. We predicted offspring counts of mice across populations by modeling the fixed effects of body mass (at the time of entrance into enclosures), sex, and a sex‐by‐mass interaction, while study, population (nested within study), and litter were included as random effects. This initial model resulted in an unexpected negative correlation between body mass and fitness in males [contrary to published findings (Franks & Lenington, 1986; Krackow, 1993)]. Therefore, we next assessed the presence of a reproductive optimum for the male data alone by performing a GLMM with the same structure (excluding sex and its interaction) above and a generalized nonlinear mixed model (GNLMM) with a second‐order polynomial term for mass and the aforementioned random effects; the GLMM and GNLMM were then compared by Akaike information criterion.

As nonlinear models can be sensitive to extreme values we also evaluated the presence of linear versus negative‐quadratic relationships between mass and fitness using a bootstrapping approach. Specifically, separate Poisson generalized linear models (GLMs) (1,000 iterations) were used to assess the influence of body mass (second‐order polynomial), and to calculate an optimum if applicable, for each sex. Assessment between linear and quadratic relationships was performed by evaluating the consistency of positive and negative values (95% CIs) of first‐ and second‐order polynomial terms for mass. The influence of extreme values is mitigated as bootstrapping utilizes random sampling with replacement, which ensures that overall patterns are not driven by individual data points. Importantly, both analysis approaches reached almost identical conclusions. All models were run in R (3.3.1) using lme4 and boot (Bates, Maechler, Bolker, & Walker, 2015; Canty & Ripley, 2016; R Core Team, 2016). Data available from the Dryad Digital Repository: http://dx.doi.org/10.5061/dryad.v3p2g.

## 3 Results

Mice weighed 15.2 ± 3.4 g (mean ± SD) range from 7.7 to 26.6 g and were sexually dimorphic with females weighing 14.3 ± 3.0 g and males weighing 17.1 ± 3.4 g (*t* test; t_67_ = −4.22, *p* < .0001). Offspring counts per mouse ranged from 0 to 109 with males producing more (36.1 ± 29.0) pups than females (12.0 ± 11.0; Wilcoxon; W = 661, *p* < .0001) as expected based on the 2:1 sex ratio.

**Figure 1:**
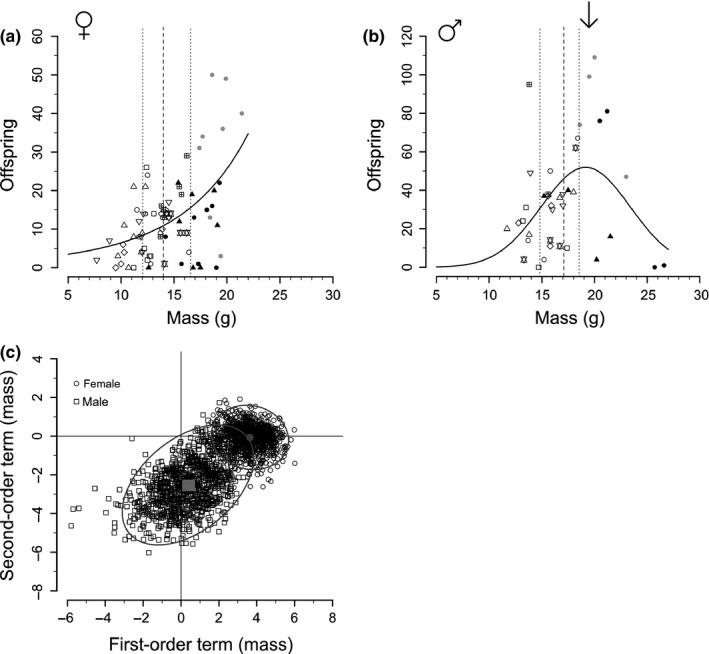
Influence of body mass on fitness. (a) For female mice, body mass is positively correlated with fitness. (b) For males, there is a negative‐quadratic relationship with an optimal mass (arrow) for fitness. For (a,b) points represent individuals, grouped by population (shapes) from three studies (colors; S1: white/open. S2: black, S3: gray), solid lines indicate best fits, while vertical lines represent medians and quartiles. (c) Different patterns between females and males are demonstrated by first‐ and second‐order polynomial coefficients of mass from bootstrap GLMs. For females, first‐order terms are consistently positive, while second‐order straddle zero, suggesting a positive relationship between fitness and mass. For males, first‐order terms span zero, while second‐order terms are negative, suggesting a negative‐quadratic relationship. Gray centers demark mean values, and ellipses indicate 95% CIs

Female fitness increased with increasing body mass (GLMM; *Z* = 2.44, *p* = .015; Figure 1a; Table 1A), while this relationship differed in males (GLMM; *Z* = −6.60, *p* < .0001). Male body mass had a negative‐quadratic relationship with high fitness possessing an optimal mass, as indicated by the GLMM having essentially no support (ΔAIC = 24.9) relative to the GNLMM (Figure 1b; Table 1B). Moreover, bootstrap models indicate a positive (95% CI: 1.80, 5.09) first‐order polynomial term for mass in females and negative (95% CI: −5.25, −0.21) second‐order mass terms in males (Figure 1c); these bootstrapping results are indicative that overall patterns are not driven by extreme points (e.g., the heaviest mice).

**Table 1:** Body mass and fitness model results

(A) Influence of body mass on fitness by sex. GLMM with Poisson distribution and logarithmic link (intercept at 15.23 g; 113 mice born in 60 cages, founded 10 populations nested in three studies)		
Random effects	Variance	Std. deviation
Study	0.2789	0.5281
Population	0.0384	0.1960
Litter	0.5273	0.7261

Fixed effects	Estimate	Std. error	*Z* value	Pr(>|*z*|)
Intercept	2.6354	0.3453	7.63	<0.0001
Mass (g)	0.0622	0.0255	2.44	0.0148
Sex (male)	1.0206	0.0899	11.35	<0.0001
Mass × sex (male)	−0.1558	0.0235	−6.60	<0.0001

(B) Influence of body mass on male fitness. GNLMM with Poisson distribution and logarithmic link (38 mice born in 33 cages, founded 10 populations nested in three studies)		
Random effects	Variance	Std. deviation
Study	0.3045	0.5518
Population	0.4262	0.6528
Litter	1.0437	1.0216

Fixed effects	Estimate	Std. error	*Z* value	Pr(>|*z*|)
Intercept	3.4038	0.5830	5.84	<0.0001
First‐order term (mass)	−5.2139	1.3775	−3.79	0.0002
Second‐order term (mass)	−3.6361	0.8205	−4.43	<0.0001

## 4 Discussion

We demonstrate a positive relationship between body mass and female fitness and a negative‐quadratic relationship in males. The positive relationship in females likely indicates larger females have higher fecundity, a pattern also seen within other rodents such as deer‐mice (*Peromyscus* sp.*)* and voles (Arvicolinae) (García‐Navas, Bonnet, Bonal, & Postma, 2016; Myers & Master, 1983), although alternative hypotheses such as differential resource control cannot be eliminated. As sexual selective forces are largely present within enclosures it is likely these forces which influence the observed optimum in male mass; for example, it is possible there is an optimal mass for winning agonistic contests, perhaps balancing strength/agility, or that females prefer to mate with males of intermediate size. These observations suggest that house mouse body size is, at least partially, constrained by male sexual competition and that the simple paradigm of “bigger is better” in regard to sexual selection is not applicable to this species.

We are able to assess the possibility of male sexual selection constraining body size because our study design and species selection control for three of the four costs of large body size suggested by Blanckenhorn (2000). By releasing all mice as adults we control for juvenile viability selection and by excluding predators and most parasites, while providing ample access to food/water we greatly reduce the pressure of adult viability selection. Likewise, the proposed cost of “late reproduction” is thought to be of primary importance in species with low encounter rates or with constrained mating periods, neither of which apply to house mice (Singleton & Krebs, 2007). The elimination of three of the four characterized costs of large body size allows us to conclude that stabilizing selection on body size, due to male–male competition, female mate choice, or a combination of the two is sufficient to constrain house mouse body size—an intriguing finding in a polygynous mammal.

Previous studies investigating relationships between fitness and body mass of house mice in semi‐natural enclosures have relied on dominance status as a proxy for fitness. A study of 32 mice found a marginally significant trend that “fitness‐rank,” based on social dominance, was positively correlated with male mass, but not female mass (Krackow, 1993), while another larger study found positive relationships for “dominance‐rank” and body mass in both sexes (Franks & Lenington, 1986). Importantly, neither study considered an optimal mass nor directly assessed fitness. One caveat concerning our study is that the analyses were limited to un‐manipulated control mice, who were cohoused with treatment individuals. Although this asymmetry in individual quality could influence the observed relationships, we find this unlikely as control mice were primarily in competition with each other, and gradients of individual quality are the norm in nature. In light of this caveat, it should be acknowledged that a study designed to directly test the influence of body mass on mouse fitness within semi‐natural enclosures considering nonlinear selection would be definitive; however, the results provided here are unique and illuminating on the selective forces shaping the evolution of body size.

Although the notion that a causal relationship between male body size and success in acquiring mates leads to increased body size in both sexes is well supported in vertebrates (Fairbairn, 1997), it may not explain patterns of SSD wherein maximal male size is not optimal. Typically it is assumed that sexual selection for increased size in males is counteracted by natural selection (e.g., predation, interspecific competition); however, the presence of an optimal body mass in semi‐natural enclosures with high levels of sexual selection (and reduced levels of natural selection) indicates house mice may be an exception to this rule. Broadly, our results support that fecundity selection in females may be a primary selective agent for large body size, but question the extent to which larger body size in males is universally beneficial in the context of sexual selection. Moreover, based on the observation herein, that larger females have more offspring when natural selection is relaxed, perhaps instead of asking “why are males relatively large?” we should ask “why are females small?.”

## Conflict of interest

None declared.

## Author Contributions

J.S.R., J.W.C., A.C.N, and W.K.P., designed the study. S.M.G., S.M., conducted enclosure studies. S.M.G. and L.S.C., assessed parentage. L.C.M. curated data. J.S.R and D.H.C. analyzed data. J.S.R. and W.K.P. wrote the manuscript.
